# fMRI Evidence of ‘Mirror’ Responses to Geometric Shapes

**DOI:** 10.1371/journal.pone.0051934

**Published:** 2012-12-14

**Authors:** Clare Press, Caroline Catmur, Richard Cook, Hannah Widmann, Cecilia Heyes, Geoffrey Bird

**Affiliations:** 1 Department of Psychological Sciences, Birkbeck College, London, United Kingdom; 2 Wellcome Trust Centre for Neuroimaging, University College London, London, United Kingdom; 3 Department of Psychology, University of Surrey, Guildford, United Kingdom; 4 Department of Psychology, City University London, London, United Kingdom; 5 All Souls College, University of Oxford, Oxford, United Kingdom; 6 Institute of Cognitive Neuroscience, University College London, London, United Kingdom; University of Bologna, Italy

## Abstract

Mirror neurons may be a genetic adaptation for social interaction [1]. Alternatively, the associative hypothesis [2], [3] proposes that the development of mirror neurons is driven by sensorimotor learning, and that, given suitable experience, mirror neurons will respond to any stimulus. This hypothesis was tested using fMRI adaptation to index populations of cells with mirror properties. After sensorimotor training, where geometric shapes were paired with hand actions, BOLD response was measured while human participants experienced runs of events in which shape observation alternated with action execution or observation. Adaptation from shapes to action execution, and critically, observation, occurred in ventral premotor cortex (PMv) and inferior parietal lobule (IPL). Adaptation from shapes to execution indicates that neuronal populations responding to the shapes had motor properties, while adaptation to observation demonstrates that these populations had mirror properties. These results indicate that sensorimotor training induced populations of cells with mirror properties in PMv and IPL to respond to the observation of arbitrary shapes. They suggest that the mirror system has not been shaped by evolution to respond in a mirror fashion to biological actions; instead, its development is mediated by stimulus-general processes of learning within a system adapted for visuomotor control.

## Introduction

Mirror neurons discharge when a monkey executes an action and when it passively observes a similar action. They have been found in ventral premotor cortex (PMv), area F5 [4], [5] and rostral inferior parietal lobule (IPL), area PF [6], [7]. Since their initial discovery, mirror neurons responsive, not only to object-directed, but also to pantomimed or intransitive actions, have been discovered in F5 [8]. Mirror neurons have also been reported in F5 that appear tuned to the sight of actions executed with tools (e.g. grasping with pliers [9]) and to the sounds associated with actions (e.g. plastic crumpling) [10].

Evidence consistent with the claim that humans also have populations of neurons with mirror properties (supporting mirror ‘representations’ of action) can be obtained from studies using functional magnetic resonance imaging (fMRI). These show that areas of the human cortex, homologous to those where mirror neurons have been found in monkeys, are active when we observe and execute similar actions [11], and show characteristic patterns of adaptation. Specifically, these cortical areas are less active where an action event (‘A’; either the observation or execution of an action) is preceded by a similar action event (AA) than when preceded by a dissimilar action event (BA). Importantly, this adaptation is observed both when the two events are experienced within-modality (A_exe_−A_exe,_ or A_obs_−A_obs_) or across modalities (A_obs_−A_exe_, or A_exe_−A_obs_). For example, in a study where participants either observed or executed a precision grip or ring pulling action in successive trials, there was less PMv activation when ring pulling observation was preceded by ring pulling execution than when it was preceded by precision grip execution [12] (references to PMv include BA44, a posterior portion of the inferior frontal gyrus, because it is thought to be the human homologue of monkey premotor region F5). This crossmodal (execution-observation and observation-execution) adaptation effect has been replicated in PMv [13] and also reported in IPL [14]. It provides evidence that common neural populations are active during the observation and execution of the same actions, suggestive of mirror representations of action: Whereas repeated activation of a common ‘mirror’ population will result in a decline in its responsivity, successive activation of independent sensory and motor populations will not [15].

According to the associative hypothesis [2], [3], mirror neurons acquire their characteristic matching properties through sensorimotor learning. At birth, sensory neurons in the superior temporal sulcus and elsewhere in the cortex are weakly and unsystematically connected to motor neurons; for example in PMv. During infancy, individuals watch their own actions and are imitated by others [3], [16]. Both self-observation and being imitated cause correlated (i.e. contiguous and contingent) activation of sensory neurons and motor neurons that code similar actions. This correlated activation selectively strengthens connections between those sensory and motor neurons encoding similar actions (associative learning), giving the motor neurons ‘mirror’ properties, i.e. they discharge, not only when an action is executed, but also, by virtue of their connections with sensory neurons, when similar actions are observed. This account assumes that mirror neuron development is relatively unconstrained, as it is mediated by domain general processes of learning. Given the appropriate sensorimotor experience mirror neurons can emerge that respond to different actions in the observe and execute conditions (so-called ‘logically related’ mirror neurons [4]) or to arbitrary sensory stimuli (e.g. the sight of actions executed with tools or action sounds [9], [10]). This contrasts with the dominant view in the literature, which suggests that mirror neurons are a genetic adaptation for social interaction [1]; that mirror neurons have been ‘programmed’ by evolution to promote action understanding, or other social cognitive functions.

**Figure 1:**
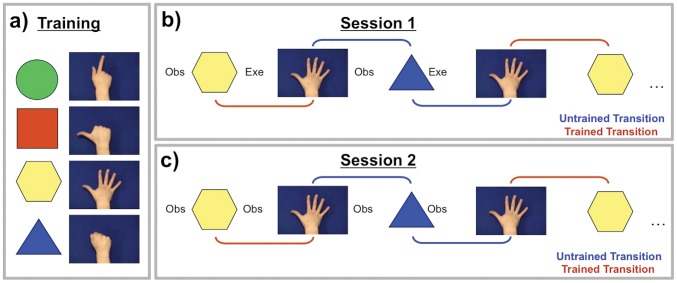
Schematic illustration of the experimental design. a) An example set of trained shape-response mappings. The relationship between shape and action type was held constant for a given participant throughout training, but was varied between participants. b) Illustration of a sequence of trials in Session 1. Observation of shapes alternated with execution of actions. A trial was ‘trained’ if preceded by an event type with which it had been paired during training, and ‘untrained’ if preceded by a different event type. c) Illustration of a sequence of trials in Session 2. In this session, observation of shapes alternated with observation of actions, and ‘trained’ trials were those in which, if training induced *mirror* representations to respond to arbitrary shapes, both the preceding and current events should activate the same motor representation. See also Table 1.

**Figure 2:**
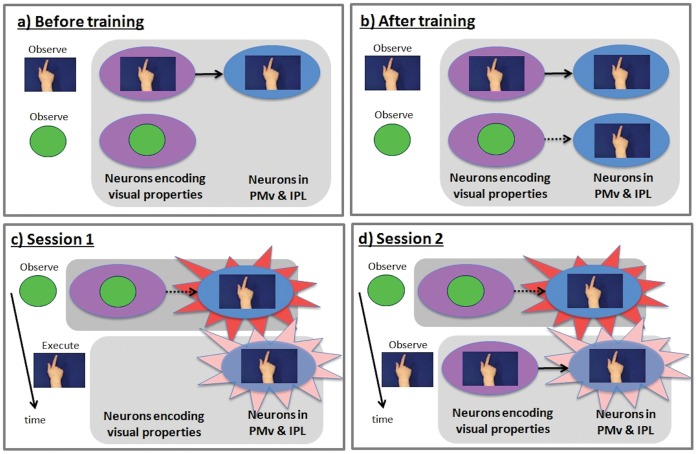
Schematic representation of the fMRI adaptation logic. Note that although reference is made to mirror *neurons*, fMRI data is driven by populations of neurons. Purple ovals denote populations of sensory neurons encoding visual properties of stimuli; blue ovals denote populations of motor neurons responsible for action execution. a) Before training, motor neurons are activated by observation of actions (top) but not by observation of shapes (bottom). These cells are therefore mirror neurons b) Training where participants respond to each arbitrary geometric shape with a distinctive action establishes novel excitatory links (broken arrow) between neurons encoding sensory properties of each shapes and motor neurons encoding the trained action. c) Session 1. Adaptation from shape observation to action execution (signified by paler flash on right), and vice versa, shows that, as a result of training, the shapes activate neuronal populations with motor properties. d) Session 2. Adaptation from shape observation to action observation, and vice versa, shows that shape and action observation activates common neuronal populations; i.e. cells with *mirror* properties. Session 2 adaptation would not have occurred if experimental training had linked visual neurons with i) purely motor neurons, ii) canonical neurons, or iii) logically related mirror neurons. The training must have linked neurons encoding the sensory properties of the geometric shapes with neurons that were already encoding both sensory and motor properties of action, i.e. congruent mirror neurons.

If the associative account is correct, it should be possible for mirror neurons in classic mirror areas (e.g. PMv and IPL) to become connected through sensorimotor learning, not only to visual neurons that code actions, but also to visual neurons that code non-action stimuli, such as geometric shapes. Connections of this kind would yield mirror neurons that are selectively responsive to the execution and observation of a particular action and to the observation of a shape that has been associated with performance of that action. In order to test this hypothesis, participants were given sensorimotor training in which they were required to perform distinct hand actions in response to different geometric shapes (see Figure 1a). It is difficult to measure the activity of single neurons in humans, and so in the current study fMRI adaptation was used to measure the activity of populations of neurons with mirror properties. After training, in the first fMRI session participants experienced runs of events in which shape observation alternated with action execution. We compared the blood-oxygen-level-dependent (BOLD) signal in ‘trained trials’, where the event was immediately preceded by an event with which it had been paired during training, and ‘untrained trials’, when it was preceded by an event with which it had not been paired (see Figure 1b). If sensorimotor training induces populations of neurons in mirror areas encoding motor properties of actions to respond to geometric shapes (see Figure 2), one would expect a lower BOLD signal in trained than in untrained trials (due to BOLD adaptation).

**Table 1:** An example of 16 trials in a block, categorised according to their Transition Type and Trial Type with respect to the previous trial.

Stimulus	Transition Type	Trial Type
Hexagon	N/A	N/A
Splay	Observe-Execute	Trained
Triangle	Execute-Observed	Untrained
Splay	Observe-Execute	Untrained
Hexagon	Execute-Observed	Trained
Fist	Observe-Execute	Untrained
Triangle	Execute-Observed	Trained
Splay	Observe-Execute	Untrained
Triangle	Execute-Observed	Untrained
Fist	Observe-Execute	Trained
Triangle	Execute-Observed	Trained
Fist	Observe-Execute	Trained
Hexagon	Execute-Observed	Untrained
Fist	Observe-Execute	Untrained
Hexagon	Execute-Observed	Untrained
Splay	Observe-Execute	Trained

These categorisations represent a participant who had been trained with hexagon-splay fingers and triangle-make fist mappings.

To determine whether sensorimotor learning changed the responses, not merely of populations of motor or canonical neurons, but of populations of neurons coding the matching sensory and motor properties of action (i.e. mirror representations), participants completed a second fMRI session in which shapes alternated with action observation (see Figure 1c). It should be noted that participants could not observe their actions during training. Therefore, in this session, ‘trained’ trials were those in which both the preceding and current event could activate a common neuronal population encoding the same motor representation. Since in this session both events comprise only observation (of shapes or of actions), they could only activate the same motor representation if the training had induced responses to arbitrary shapes not only in populations of *motor* neurons but in populations of neurons with *mirror* properties. BOLD adaptation between shapes and observation of actions in mirror areas would indicate that sensorimotor training induced mirror representations – populations of neurons that were already coding both observation and execution of similar actions - to respond to arbitrary geometric shapes. For example, for a participant trained to associate ‘observe hexagon’ with ‘execute splay fingers’, both the observation of a hexagon and the observation of splayed fingers should activate neural populations coding for the execution of splay fingers, as long as this population has mirror properties; i.e. it responds to both observation and execution of splay fingers.

Session 2 is crucial for the interpretation of any learning effects observed. If training altered the properties, not of populations of cells with mirror properties, but of other populations of neuron in the regions of interest (e.g. canonical neurons, motor neurons, or ‘logically-related’ mirror neurons), adaptation should not be observed in Session 2. That is, if sensorimotor training induced purely motor neurons to respond to geometric shapes, one would not expect shape and action observation to activate the same neural population during Session 2. In this case, although shape observation would activate the motor representation, observation of actions would not. Similarly, if training induces populations of canonical neurons to respond to observation of the shapes, adaptation would not be observed during Session 2, as one would again expect shape observation, but not action observation, to activate these populations. If training induced populations of ‘logically-related’ mirror neurons to respond to observed shapes, adaptation would not be expected during Session 2. Observation of the shapes could activate logically-related neurons coding for execution of action A, but, by definition, observation of action A would not. One would only expect adaptation in Session 2 if training induced populations of neurons coding for observation and execution of the *same* action (i.e. by definition, congruent mirror representations) to respond to the observation of the trained shape.

## Materials and Methods

### Participants

Twenty-one paid healthy participants took part in this study (8 male, mean age 24.0 years, standard deviation 4.4 years). All were right handed, assessed using the Edinburgh Handedness Inventory (EHI) [17]. One participant had an EHI score of 55 which is in the 1^st^ right-handed decile, all other participants had scores of 70 or greater. Participants had normal or corrected-to-normal vision, were naïve with respect to the purpose of the experiment, and all gave written informed consent. The experiment was performed with the approval of the Birkbeck Psychology Research Ethics Committee and performed in accordance with the ethical standards laid down in the 1964 Declaration of Helsinki.

### Stimuli

The action stimuli were generated by video recording each of four models (two male and two female) performing four different gestures with the right hand. The gestures were filmed from a first-person perspective and all started from a relaxed position with the hand supine on a featureless background. From this starting position the four gestures were: 1) point finger - curling the thumb, middle, ring and little fingers under the palm and extending the index finger so that it pointed; 2) splay fingers - extending all of the fingers and the thumb as far as possible away from the palm; 3) extend thumb - curling all fingers under the palm and extending the thumb to the side, and 4) make fist - curling the thumb and all fingers under the palm to make a fist. These gestures were used because they had previously been shown to produce robust adaptation when executed [18]. The shape stimuli consisted of a green circle, yellow hexagon, red square and blue triangle. Execution trials were cued by the words, ‘point’, ‘stretch’, ‘thumb’, and ‘fist’ presented uppercase, in white Helvetica font on a black background.

### Procedure

#### Training

During training participants were initially instructed in the correct execution of each of the four gestures. When the participant was consistently executing the correct gestures, they were asked to put their hand behind a screen so that it was invisible to the participant but visible to the experimenter. Participants were seated in front of a computer monitor where the shape stimuli were to be displayed. They were instructed that they would be asked to perform the appropriate gesture in response to a shape and that each of the four shapes cued one of the four gestures. They were also told that they would have to discover for themselves which gesture was appropriate for each shape. Thus, participants were not explicitly instructed about the shape-gesture mappings (e.g. they were not told to respond to green circles by pointing), or to make a particular gesture in any given trial. In the first eight training trials each of the shapes was presented twice. Subsequently, the order of shape presentation was randomized within each block. In each trial a shape was presented and the participant made a response. If the gesture was correct, the next trial was presented. If the participant executed the wrong gesture, a warning tone sounded and the word ‘Wrong!’ appeared on the screen. The same stimulus was then presented in successive trials until the participant made the correct response. Accuracy was monitored by the experimenter, who could see both the stimulus presented to the participant and the participant’s response. Eleven of the participants were trained with the following stimulus-response mappings: circle_obs_−point finger_exe_; square_obs_−extend thumb_exe_; hexagon_obs_−splay fingers_exe_; triangle_obs_−make fist_exe_ (see Figure 1a). The remaining ten participants were trained: circle_obs_−splay fingers_exe_; square_obs_−make fist_exe_; hexagon_obs_−point finger_exe_; triangle_obs_−extend thumb_exe_. Using different arbitrary pairings for different participants ensured that it could not have been pre-existing associations between the actions and shapes, rather than the training, which produced the observed effects. The initial period of training was completed a day before the scanning session and consisted of eight blocks of 150 trials each (lasting approximately one hour in total). A refresher period of training was completed immediately before the scanning session and consisted of four blocks of 150 trials.

#### Scanning procedure

All participants completed two sessions. During Session 1, shape observation alternated with gesture execution (see Figure 1b). Execution events were cued by the words, ‘point’, ‘stretch’, ‘thumb’, and ‘fist’. Participants were required to perform the gesture that corresponded to the word cue. Whether the first event involved shape observation or action execution was counterbalanced across participants. During Session 2, shape observation alternated with action observation (Figure 1c). Session 2 was structurally identical to Session 1; the only difference between them was that participants were required to execute actions in Session 1 and to observe actions in Session 2. Each stimulus (shape, word, or action video) was presented for 800 ms.

Each session was split into eight mini-blocks of 33 events (264 events in total). Each event was characterized as a trained or untrained trial with respect to the preceding event (i.e. using the methodology employed by Hamilton and Grafton [18]; see Figure 1 and Table 1. The first event in each mini-block was therefore discarded, resulting in an effective design of eight mini-blocks of 32 trials each (256 trials in total). Each mini-block comprised a factorial design with factors of Trial Type (trained or untrained), ISI (short or long), and Transition Type (observation - execution, or execution - observation). Adaptation was assessed over both short and long trial-to-trial and session timescales: ISI between stimuli was fixed at 250 ms (‘short’), or was randomly jittered between 2 and 4 seconds (mean 3 seconds, ‘long’). In addition, mini-blocks 5–8 were an exact replication of mini-blocks 1–4, enabling adaptation to be assessed during a short session (mini-blocks 1–4), and across a longer session (comparison with mini-blocks 5–8). Four repetitions of each combination of the ISI, Trial Type, and Transition Type factors made up each mini-block. The trial order within mini-blocks was randomly determined. Each mini-block contained only two examples of the four shape-gesture pairings (e.g. in a particular mini-block participants may have only observed circle and square stimuli and only executed point finger and extend thumb). This ensured that the number of specific combinations of shape and gestures presented in a mini-block did not differ between trained and untrained trials, and therefore that the opportunity to learn new associations did not differ between trained and untrained trials during the fMRI sessions. The two examples were chosen randomly in each of mini-blocks 1–4, and replicated in mini-blocks 5–8.

Participants were filmed throughout Session 1 so that the experimenter could ensure online, and subsequently offline, that they were executing 1) the actions they were instructed to perform, and 2) during execution periods only. Participants made very few errors. They omitted a cued response in 0.69% of trials, made an incorrect response in 0.39% of trials, and made an uncued response in 0.49% of trials. Given the low rate of errors, the behavioral data were not analysed further.

### Data Acquisition and Analysis

We acquired T2*-weighted echo-planar images (EPI) with BOLD contrast on a 1.5T whole-body MRI scanner (Siemens AG, Erlangen, Germany) in two sessions (TR = 2.556s, TA = 2.556s, 30 axial slices, 4 mm×4 mm×4 mm in-plane resolution) operated with a 32-channel head coil. A total of 252 volumes were collected for each of the two sessions, including 6 dummy volumes at the start of each session to allow for T1 equilibration. High-resolution T1-weighted structural scans were collected for all but one participant and were co-registered to their mean EPI images. For the participant where it was not possible to collect a T1 scan, we co-registered functional scans to the Montreal Neurological Institute (MNI) EPI template.

Data pre-processing of the EPI functional scans, including spatial realignment, unwarping, normalization to the standard MNI template, and smoothing with a 4 mm (full-width half-maximum, FWHM) Gaussian kernel, was completed using SPM8 (www.fil.ion.ucl.ac.uk/spm). The event-related fMRI data were then analysed using a linear convolution model. We included 32 regressors of interest, which were regressed against the EPI data and high-pass filtered at 128 seconds to remove low-frequency drift. These regressors were derived by convolving a canonical hemodynamic response function and its temporal derivative with delta functions representing stimulus onset of each unique combination of the levels of the factors. The factors were Trial Type (trained, untrained), Transition Type (observation - execution, execution - observation), ISI (short, long) and Session Half (miniblocks 1–4, miniblocks 5–8) factors). The resulting beta images for each condition were combined to form magnitude images as described in Steffener et al. [19]. These subject (first) level magnitude images were used to generate contrast images, which were smoothed with a 4 mm FWHM Gaussian kernel, and entered into a random effects (second-level) analysis to investigate group-level responses. All co-ordinates are reported in MNI space.

Two analyses were performed. The first was performed within regions of interest (ROIs) that corresponded to 10 mm spheres around the peak voxels within PMv and parietal cortex where previous studies have found crossmodal (observation – execution or execution – observation) action adaptation effects. These voxels were taken from the studies of Chong et al. [14], Lingnau et al. [20], Kilner et al. [12], and Press et al. [13]. We generated two ROIs; one for cross-modal effects within PMv and IFG, consisting of spheres around [−50,−2,12] [12], [−56,2,20] and [56], [4], [12] [13], and another for effects within parietal cortex; consisting of spheres around [58,−56,34] (IPL [14]), [−46,−37,27] (intraparietal sulcus [20]), and [−28,−59,46] (superior parietal lobule [20]). Significance levels within this analysis were family-wise error corrected for the ROI volume at a voxel-level of *p*<0.05 and a cluster extent threshold of four voxels. The second analysis investigated responses across the whole brain at a threshold of *p*<0.001 uncorrected, with a cluster extent of four voxels.

## Results

**Figure 3:**
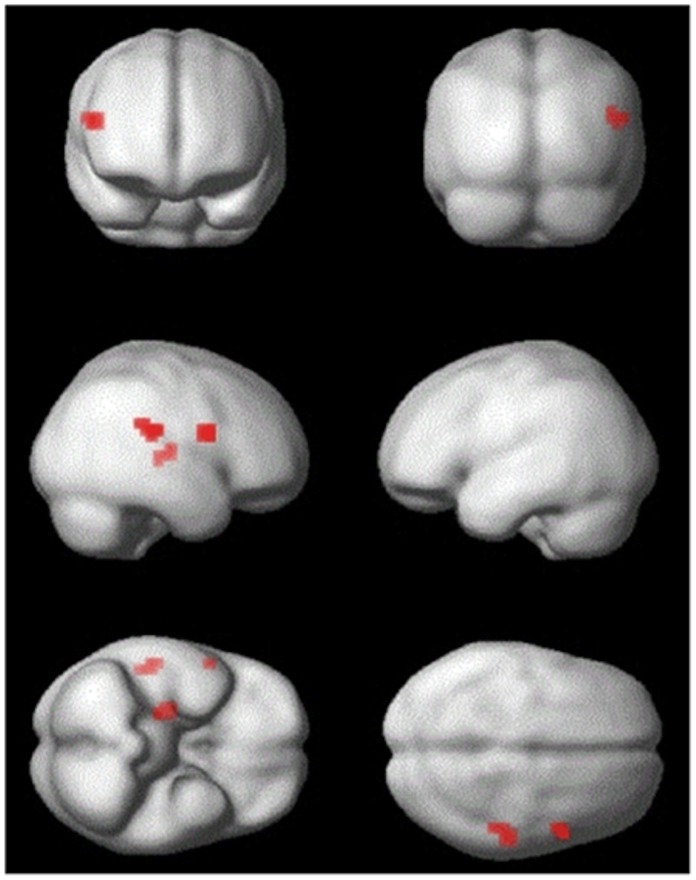
Statistical parametric maps (SPM) of the main effects of adaptation in Session 1. The SPM is thresholded for display at *p*<0.001 uncorrected with a cluster extent of 4 voxels. Results are rendered upon the smoothed average brain provided in SPM8.

fMRI adaptation was calculated by contrasting trained and untrained trials (untrained - trained). In Session 1, ‘trained’ trials were those in which the preceding event had been paired with the current event during training (Figure 1b). Thus, for a participant trained to associate ‘observe circle’ with ‘execute point’, a trained trial would consist of either observation of a circle preceded by execution of a point action, or execution of a point action preceded by observation of a circle. In ‘untrained’ trials the previous and current events had not been paired (e.g. observation of a circle following execution of a ‘fist’ action). Adaptation from shape observation to action execution, or vice versa, would indicate that as a result of training, neuronal populations responsive to action execution are also responsive to shape observation. An adaptation effect surviving family-wise error correction (FWE) was found within the PMv ROI, with a peak at [54], [8], [18], *t* = 4.5, *p*<0.05 FWE (see Figure 3). In Session 1 there were no voxels surviving FWE correction within the Parietal ROI, but there was a cluster at *p*<0.001 uncorrected, with a peak at [54,−28,22]. Peak voxels demonstrating a main effect of adaptation are reported in Table 2.

**Table 2:** All peak coordinates for the main effect of adaptation in Session 1, at *p*<0.001 uncorrected, with a cluster extent of four voxels.

x	y	z	t	Cluster size	Area
54	−28	22	5.55	9	Right inferior parietal cortex
26	−16	10	4.58	7	Right putamen
54	8	18	4.53	8	*Right inferior frontal gyrus (BA44)

* Indicates significant at *p*<0.05 FWE corrected for search volume. BA, Brodmann Area.

**Figure 4:**
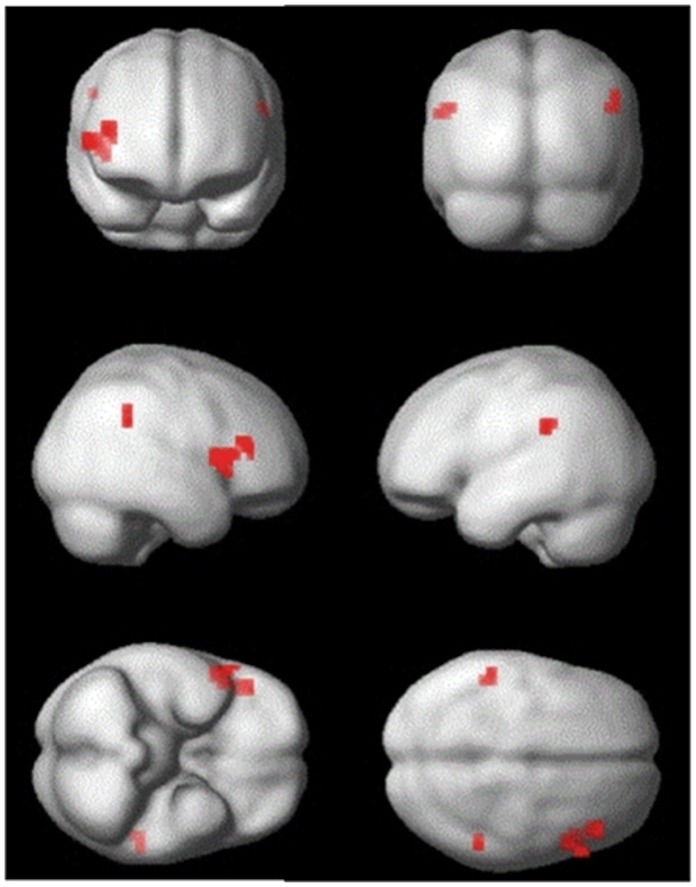
Statistical parametric maps (SPM) of the main effects of adaptation in Session 2. The SPM is thresholded for display at *p*<0.001 uncorrected with a cluster extent of 4 voxels. Results are rendered upon the smoothed average brain provided in SPM8.

In Session 2, shape observation alternated with action observation. Thus ‘trained’ trials (Figure 1c) were those in which, if training induced *mirror* representations to respond to arbitrary shapes, both the preceding and current events should activate a neuronal population coding the same motor representation. ‘Untrained’ trials were those in which the preceding and current events were not predicted to share a motor representation. Adaptation from shape observation to action observation, or vice versa, would therefore indicate that as a result of training, shape observation activates a neural population responsive to observation and execution of the same action: that is, a population of neurons with mirror properties. Adaptation effects were observed both within the PMv (peak at [54], [12], [6], *t* = 4.4, *p*<0.05 FWE) and Parietal (peak at [−54,−36,30], *t* = 4.2, *p*<0.05 FWE) ROIs (see Figure 4). Additionally, there was a right parietal adaptation effect at *p*<0.001 uncorrected, with a peak at [54,−44,38]. Peak voxels demonstrating a main effect of adaptation in Session 2 are reported in Table 3.

**Table 3:** All peak coordinates for the main effect of adaptation in Session 2, at *p*<0.001 uncorrected, with a cluster extent of four voxels.

x	y	z	t	Cluster size	Area
54	12	6	4.46	14	*Right inferior frontal gyrus (BA44)
−54	−36	30	4.24	5	*Left inferior parietal cortex
46	28	18	4.09	9	Right inferior frontal gyrus (BA45)
54	−44	38	4.02	4	Right inferior parietal cortex

* Indicates significant at *p*<0.05 FWE corrected for search volume. BA, Brodmann Area.

We also investigated whether adaptation interacted with any of the other variables (Inter Stimulus Interval (ISI), Session Half, or Transition Type). In Session 1, we found an area where adaptation was modulated by ISI within the PMv ROI, at two peak coordinates ([−58,8,26], *t* = 4.4, *p*<0.05 FWE; [−62,4,22], *t* = 4.1, *p*<0.05 FWE). At these coordinates, the adaptation effect was greater at short ISIs. Previous studies have also found greater adaptation effects at shorter ISIs [21], [22], suggesting that adaptation effects within certain regions, including left PMv, may be short-lived. There were no areas within the Parietal ROI in Session 1, or in either ROI in Session 2, where adaptation was modulated by ISI, Transition Type or Session Half.

## Discussion

The present experiment set out to test a specific prediction of the associative account of the development of mirror neurons: If mirror neurons acquire their properties through domain general processes of sensorimotor learning, it should be possible for human mirror neurons to respond to arbitrary non-action stimuli following exposure to appropriate sensorimotor contingencies. While a test of this hypothesis would ideally involve single-cell recording in humans, due to the difficulties in obtaining these data the present study utilised fMRI adaptation in order to record the BOLD signal from populations of neurons with properties consistent with mirror neurons. To test the associative hypothesis, participants were trained to execute different responses (point, splay fingers, make fist, extend thumb) to the onset of different geometric shapes (green circle, red square, yellow hexagon, blue triangle). After training participants completed two scanning sessions where the observation of either shapes or actions alternated with action execution (Session 1) or where observation of shapes alternated with observation of actions (Session 2). This procedure, particularly the inclusion of Session 2, allowed us to use the fMRI adaptation technique to provide evidence of the activation of populations of neurons with mirror properties.

The results confirmed the prediction of the associative account. When shape observation alternated with action execution (Session 1), adaptation was observed in PMv: for both shapes and actions, the BOLD signal associated with an event was lower when that event was preceded by an event with which it had been paired during training than when it followed an untrained event. PMv is an area in which mirror neurons have been found in the macaque [4], [5], and where adaptation effects indicative of populations of neurons with mirror properties have been found when humans observe and execute similar actions [12], [13]. Therefore, consistent with the associative account of the development of mirror neurons [2], [3], the findings from Session 1 suggest that the sensorimotor training at the beginning of the experiment strengthened connections between visual neurons coding the colour and/or shape of geometric stimuli and neurons in a classical mirror area encoding the motor properties of action.

The above interpretation of the results of Session 1 is supported and strengthened by the findings from Session 2: When observation of shapes alternated with observation of actions, we found an adaptation effect in PMv and in another classical mirror area, IPL [6], [7]. For both shapes and actions, the BOLD signal associated with observing an event was lower when that event was preceded by observation of an event with which it shared a motor representation, through training, than when it was preceded by observation of an event with which it did not share a motor representation. Even without data from Session 1, these results provide evidence that training induced populations of neurons with mirror properties, rather than any other population, to respond to geometric shapes. The adaptation effects seen in Session 2 show that common neural populations were coding the visual properties of the actions used during training and also the shapes. These populations could not have acquired the capacity to map visual properties of the shapes onto visual properties of the actions during training, because participants were not allowed to see their own actions at any stage in the experiment. Therefore, the adaptation effect in Session 2 implies that the sensorimotor training at the beginning of the experiment induced mirror representations – populations of neurons in the PMv and IPL that were already coding both observation and execution of similar actions – to respond to arbitrary geometric shapes.

These results are consistent with research showing that activation in mirror areas varies with expertise [23] and training [24], [25], and with previous reports that sensorimotor learning can induce [26], [27], enhance [28], abolish [29], [30], [31], [32], [33] and even reverse [34], [35], [36] ‘mirror effects’, i.e. effects of action observation on overt behaviour, motor evoked potentials (MEPs), and BOLD responses in mirror areas. For example, Catmur et al. [35] showed that sensorimotor training can reverse a ‘Fadiga effect’ [37] in which transcranial magnetic stimulation (TMS)-induced MEPs are larger in the index finger muscle when observing index finger than little finger actions, and larger in the little finger when observing little finger than index finger actions. After incompatible sensorimotor training, in which participants executed little finger actions when observing index finger actions, and vice versa, observation of index finger actions induced greater MEPs in little finger muscles, and observation of little finger actions induced greater MEPs in index finger muscles. Indeed, in a closely-related study Petroni et al. [27] found that, following training where an arbitrary shape cue was paired with an action, passive observation of the shape cue activated motor representations of action, presumably via mirror areas. Evidence of plasticity observed in previous studies of expertise and sensorimotor training, and in the present study, accords with the predictions of the associative hypothesis [2], [3]. Moreover, the present findings contribute to growing evidence that the effects of sensorimotor training modulate mirror effects by modifying mirror representations in classical mirror areas [34], [36].

Contrary to the view that mirror neurons were specifically designed [1] or ‘canalised’ [38] by genetic evolution to mirror observed actions, the associative account implies that there is nothing intrinsically ‘mirror’ about mirror neurons. The associative account predicts that sensorimotor learning can readily cause populations of motor neurons, responsible for the performance of both transitive and intransitive actions [8], to become associated with the observation of similar actions (strictly and broadly congruent mirror neurons [5]), dissimilar observed actions (logically related mirror neurons [4]), actions performed with tools (tool-use mirror neurons [9]), characteristic action sounds (audiovisual mirror neurons [10]), and action-appropriate objects (canonical neurons [39]). For example, audiovisual mirror neurons respond to action sounds such as plastic crumpling and metal striking metal [10]. Under the associative account, these cells acquire their properties through experience of performing actions while hearing these sounds, but are harder to accord with evolutionary hypotheses [40]. The present study confirms that geometric shapes - a class of arbitrary non-action stimuli that have neither the morphological or dynamic properties characteristic of body movements - should be included in the list of sensory stimuli that can elicit excitation of populations of cells with mirror properties following contingent sensorimotor experience. The present findings suggest that differences in response patterns among these different neurons (broadly and strictly congruent mirror neurons, logically-related mirror neurons, tool-use mirror neurons, audiovisual mirror neurons, canonical neurons, geometric-shape mirror neurons) may not reflect different cell types, but rather the fact that motor neurons may become associated with different eliciting stimuli. Consequently, the particular ‘class’ of observed stimuli that causes the cell to fire may not be a normative, intrinsic property of the cell itself, but may instead be a consequence of the individual’s learning history [41], [42].

An alternative interpretation of the present data could be advanced whereby the matching property of mirror representations has been designed by evolution to promote action understanding or social interaction, but these representations will additionally encode arbitrary stimuli following appropriate learning. There are at least two versions of this hypothesis. First, mirror representations are present at birth [1], but these representations are not buffered against becoming responsive to other stimuli through learning. Second, the development of mirror representations may be incompletely canalised, such that these representations are acquired more readily in this population of cells, but not to the exclusion of other response properties. These hybrid models, along with a wide range of additional recent hybrids, represent interesting potential advances on the two accounts contrasted historically and in the present study, but there is currently no independent data to support them.

fMRI adaptation effects provide evidence that common neural populations code both events [15], [43]. However, the neural mechanism underlying BOLD adaptation at the single-cell level is a topic of debate. While it may reflect reduced firing rate of single cells, it may also reflect firing of fewer cells, or faster, more efficient, processing of the stimulus and its ‘downstream’ effects (the ‘facilitation model’, [15], [43]). Here, we have used BOLD adaptation solely as an index of neural specialisation, i.e. to identify the presence of a common neural population encoding actions and events with which they have been paired in training, rather than to investigate the mechanism by which that reduction occurs (see also [44]). Therefore, the interpretation of our BOLD adaptation results is appropriate irrespective of whether individual mirror neurons show reduced firing rates with repeated stimulus presentation.

### Alternative Accounts

We have argued that the sensorimotor training completed before the scanning session induced associations between sensory populations of neurons encoding geometric shapes, and populations of neurons with mirror properties which were already responsive to the observation and execution of actions. In this section, we will consider a number of alternative accounts, and explain why we believe these to be unlikely explanations of our findings.

First, rather than reflect novel *sensorimotor* associations, it could be argued that the observed adaptation effects could be caused by associations between the sensory descriptions of shapes and actions. Such *sensory-sensory* associations might allow shape observation to activate motor representations (Session 1) indirectly, via sensory representations of action, and sensory descriptions of action (Session 2) directly. However, this account is unlikely. Crucially, participants’ hands were occluded from view throughout both training and scanning phases. Consequently the sensory descriptions of the shapes and actions were never paired (i.e. temporally contiguous and contingent), thus the necessary conditions for sensory-sensory associative learning were not met. A related alternative account might hold that if participants imagined the sensory consequences of their actions during training [45] this may have yielded enough pairings to produce weak sensory-sensory associations. However, for either of these alternative accounts to be true, it would have to be the case that execution of the action caused the sensory description of that action to be activated. Thus, the sensory description of the shape would activate populations of neurons coding for both action execution and the sensory description of that action i.e. populations of neurons coding for mirror representations.

Second, classical mirror areas do not only contain mirror representations of action; they also contain substantial populations of canonical neurons [39], [46], thought to mediate object affordances. Could the adaptation effects observed be the product of canonical populations acquired long before the experiment? While geometric shapes lack the three-dimensional properties of the objects on which transitive actions are typically performed, it is possible that geometric shapes prime object properties – for example, a circle may activate neurons sensitive to the properties of spheres. However, this account is also implausible. Our training regime paired actions with shapes in a way that was both arbitrary with respect to any priming of this sort, and different between participants. For example, the observation of a circle (or sphere) no more ‘affords’ pointing than the observation of a square (or block), and does not do so more for the participants trained with this pair compared to those trained with the circle-splay finger pairing. The reliable motor activations during shape observation, seen in both sessions, are therefore unlikely to be the product of canonical neurons shaped before the experiment. Moreover, populations of canonical neurons could not produce the adaptation seen in Session 2 to the alternating observation of shapes and actions, as canonical neurons, by definition, are not responsive to the sight of actions.

Third, it has been well-established using behavioural [47] and neurophysiological methods [48], [49] that both human and non-human animals show associative learning when they experience a contiguous and contingent relationship between an arbitrary stimulus and a motor response. It could be argued that the adaptation effects seen in Session 1 are the product of novel stimulus-response associations between the sensory representations of the geometric shapes and purely motor (i.e. non-mirror) representations. However, the results of Session 2 cannot be explained by this type of learning. Crucially, in Session 2, common motor populations were excited by both shape and action observation. This finding demonstrates that shapes were associated with motor populations that were already responsive to the sight of the same actions; i.e. mirror representations of action. Interestingly, previous research has found effects of associative learning in dorsal premotor cortex (PMd) [48], [49], whereas mirror neurons have been found in the monkey PMv. However, the results of the present study, together with the results of Cisek and Kalaska [50] who found neurons with mirror properties in PMd, indicate that the loci of mirror representations and of training effects are not, in fact, dissociable (see also [36]).

Fourth, could the adaptation effects observed be due to adaptation of neurons coding for verbal or semantic representations of action, rather than populations of neurons with mirror representations? Such representations are thought to depend on regions of IFG [51], [52]. Under this interpretation, shapes become associated not with mirror representations but with verbal or semantic representations of the trained actions. Thereafter, neural populations encoding semantic or verbal descriptions of action may be excited both during observation and performance, via pre-experimental learning, and also during the observation of shapes, through associations acquired during training. However, if a population of neurons fires during the execution and observation of the same action - as is required in order to explain the adaptation in Session 2 - then that population meets the functional definition of a mirror representation, irrespective of whether that population also responds to verbal descriptions/semantic representations of actions. A ‘semantic account’ is therefore an extension of, and perfectly compatible with, the idea that the properties of mirror representations have been changed by training. Elucidating the nature of the information encoded by populations of neurons firing during observation and execution of action in different regions is a research aim common to all those researching the functions and origins of the human mirror system (e.g. [53]). However, the observation that regions containing mirror neurons are considered to be the same as those involved in language processing is precisely what has prompted some authors to speculate that mirroring and language processes are closely related [54].

In conclusion, the results of the present study is consistent with the idea that mirror neurons are not ‘specialists’; i.e. they have not been shaped by evolution [1] or ‘canalised’ [38] to respond in a mirror fashion to biological actions. One would expect the development of an adaptation to be buffered against such short-lived variations in the environment [3], [55], [56]. In contrast, evidence that populations of cells with mirror properties can become responsive to static, non-biological stimuli after a relatively short training period supports the associative account of the origin of mirror neurons. This account proposes that the development of mirror neurons is mediated by stimulus-general processes of learning within a system that is adapted for basic visuomotor control. Under this account, mirror neurons may contribute to social interaction, but they are not specialised for this role.
